# Reproductive Control in Dogs with Emphasis on Anti-GnRH Immunocastration and Its Behavioral Effects

**DOI:** 10.3390/vetsci13010005

**Published:** 2025-12-20

**Authors:** María José Ubilla, Manel Lopez-Bejar, Daniela Siel, Leonardo Sáenz

**Affiliations:** 1 Escuela Medicina Veterinaria, Facultad de Medicina Veterinaria y Agronomía, Universidad de las Américas, Avenida Manuel Montt 948, Providencia, Santiago 7500000, Chile; mubillac@udla.cl; 2 Programa de Doctorado en Producción Animal, Universitat Autònomade Barcelona, 08193 Bellaterra, Spain; 3 College of Veterinary Medicine, Universitat Autonoma de Barcelona, 08193 Bellaterra, Spain; manel.lopez.bejar@uab.cat; 4 Escuela de Medicina Veterinaria, Facultad de Medicina y Ciencias de la Salud, Universidad Mayor, Camino La Pirámide 5750, Santiago 8580835, Chile; 5 Centro de Biomedicina, Universidad Mayor, Camino La Pirámide 5750, Santiago 8580835, Chile; 6 Laboratorio de Vacunas Veterinarias, Facultad de Ciencias Veterinarias y Pecuarias, Universidad de Chile, Santiago 8820808, Chile

**Keywords:** dog overpopulation, immunocastration, GnRH vaccine, behavioral effects, surgical sterilization, animal welfare

## Abstract

Dog overpopulation poses serious challenges to public health, animal welfare, and environmental sustainability. While surgical castration remains the most commonly used method for controlling reproduction in dogs, it carries risks and limitations, including surgical complications and long-term behavioral or physiological side effects. This review examines the potential of immunocastration—vaccination targeting gonadotropin-releasing hormone (GnRH)—as an alternative approach. Immunocastration has been shown to suppress reproductive hormones, reduce gonadal function, and decrease behaviors such as roaming, aggression, and sexual mounting in dogs. The review also includes evidence from other species (e.g., pigs, cattle, and horses) to contextualize effectiveness and welfare implications. While behavioral effects are more variable and less frequently studied than physiological outcomes, recent findings suggest immunocastration may have fewer negative emotional side effects (e.g., anxiety and stress-related behaviors) than surgical sterilization. The review highlights the mechanisms, applications, and behavioral outcomes of immunocastration, including its reversibility, cost-effectiveness, and suitability for mass implementation. Overall, immunocastration offers a promising addition to dog population management strategies, particularly in regions with limited surgical infrastructure. Future research should focus on standardizing vaccine protocols and assessing long-term behavioral and welfare outcomes in diverse dog populations.

## Simple Summary

Dog overpopulation is a global issue affecting public health, the environment, and animal welfare. Many free-roaming dogs live in cities, contributing to the spread of diseases, traffic accidents, and environmental contamination. Traditional control methods, like surgical castration or euthanasia, can be costly, invasive, or socially unacceptable. Immunocastration—a type of vaccination that prevents reproduction by targeting reproductive hormones—offers a promising, more humane alternative. This approach avoids surgery, reduces unwanted behaviors like aggression or roaming, and can be applied on a large scale. This review explores how immunocastration works, its effects on dogs’ behavior and health, and how it compares to traditional sterilization. It also reviews evidence from other species, including pigs, cattle, and horses, where immunocastration has been used successfully. The findings show that this method can help reduce dog overpopulation more ethically and sustainably, especially in areas with limited veterinary resources. By better understanding the benefits and limitations of immunocastration, veterinarians and policymakers can make more informed decisions to promote animal and public health.

## 1. Introduction

The increasing coexistence between humans and pets reflects a complex and multidimensional social challenge, encompassing impacts on human quality of life, public health, environmental integrity, animal welfare, etc. [1,2]. Globally, the dog population is estimated to range between 700 [3,4] and 900 million [5], with approximately 75% of these dogs roaming and reproducing freely [6,7]. Although most of these dogs have owners [8,9], fewer than 10% are truly ownerless. However, many are allowed to roam freely, reflecting patterns of irresponsible ownership rather than the absence of owners [10,11]. Each year, between 6 and 8 million dogs and cats enter shelters, many of which are overwhelmed and forced to limit admissions or resort to euthanasia. It is estimated that 2.7 million healthy, adoptable animals are euthanized annually in shelters [12].

Free-roaming dogs often suffer from hunger, illness, and lack of care [13,14] and frequently approach human communities seeking shelter [14]. Their unsupervised presence contributes to overpopulation [7,15,16], supported by high adaptability, year-round reproductive capacity, and trophic opportunism [6]. Furthermore, domestication has likely enhanced dietary flexibility [17,18]. Thorne [17] noted that food remnants from human settlements served as a primary food source for early domestic dogs, promoting trophic opportunism. Similarly, Bhalla et al. [19] suggest that garbage acts as a secondary food source for owned free-roaming dogs and as a primary resource for truly stray populations. Several studies have shown that the presence of dogs in urban areas declines significantly when food waste is secured or properly managed [8,20,21].

Unsupervised and ownerless dogs pose multiple challenges in urban settings, including the transmission of zoonotic diseases, attacks, and bites [13]. Dogs are vectors for diseases such as rabies [20,22,23,24], leptospirosis, trichinosis, dirofilariasis, echinococcosis, hydatidosis, larva migrans, brucellosis, and scabies, among others [25,26]. Other public health concerns include physical attacks, bites, and environmental contamination with garbage and feces [19,25,27,28,29,30,31,32,33,34,35,36,37]. According to the World Health Organization [3], while there are no global estimates of dog bite incidence, studies suggest that tens of millions of injuries occur annually, with approximately 4.5 million people bitten by dogs each year in the United States alone [3]. In low- and middle-income countries, the burden of dog bites is even higher. In India, Sudarshan et al. [38] estimated that around 12 million people are bitten by free-roaming dogs each year. Similarly, Calderón et al. [39] reported an annual incidence rate of 105.6 dog bites per 100,000 inhabitants in Chile, where most victims were children aged 1–14 years and injuries were mainly located on the lower extremities. Comparable age-related patterns have also been described in Ecuador. Consequently, dog bites are recognized as a significant global public health problem [3], with serious physical [40], fatal [41], and psychological [42,43] consequences for humans.

Environmental contamination is another critical issue linked to the abundance of free-roaming dogs. Fecal matter and urine contribute to bad odors, attract flies and rodents, and pose a sanitation risk [13]. The accumulation of dog waste in urban areas represents an important source of environmental pollution and potential transmission of zoonotic pathogens [25]. Additionally, free-roaming dogs are involved in road accidents [2,25,26,31,44], are linked to the transmission of diseases to livestock and livestock predation [7,45,46], and pose a major threat to biodiversity [4,5,45,47,48,49,50,51,52,53].

Given these complex and overlapping impacts, the need for effective, ethical, and sustainable methods for dog population control is urgent. Among non-surgical strategies, immunocontraception—particularly GnRH-based immunization—offers promising outcomes in terms of efficacy, safety, animal welfare, and public acceptability. This review presents a comprehensive synthesis of immunocastration in dogs, examining its physiological and behavioral effects and assessing its potential role in veterinary public health programs.

## 2. Reproductive Control in Dogs: Approaches and Considerations

Controlling dog reproduction is essential to address overpopulation and its associated health and welfare problems. Various methods have been developed and applied in veterinary practice, ranging from surgical sterilization to hormonal and immunological approaches. The choice of strategy depends on biological, ethical, and socioeconomic factors, as well as the feasibility of implementation in different settings.

Among the strategies implemented to control domestic dog overpopulation worldwide, mass culling, educational campaigns, surgical sterilization, and non-surgical contraception have been the most common approaches. Mass culling has been employed in various countries primarily to control infectious zoonotic diseases such as rabies [54] and leishmaniasis [55]. However, this practice has proven both ineffective and socially unacceptable. Moreover, for ethical, ecological, and economic reasons, the large-scale elimination of animals is no longer considered an acceptable method for disease control [56]. Thus, public policy decisions must be evidence-based, aiming to effectively manage dog and cat populations in urban areas and reduce zoonotic risks [44].

Educational campaigns promoting responsible pet ownership have also been developed [57,58,59], although effectiveness can be limited [60] and often depends on cultural factors [57], campaign design, and proper evaluation. Nevertheless, such initiatives can contribute meaningfully to improving the welfare of companion dogs [60].

In Latin America, countries such as Chile reflect similar challenges. Several studies have described canine demographics in Chile [8,61,62,63,64,65,66]. In 2006, it was estimated that 214,933 free-roaming dogs lived in the Metropolitan Region alone [8,66]. By 2015, the population of owned dogs had reached approximately 3.5 million, with 29% reported to roam public spaces without supervision [67]. Later data showed that six out of ten Chilean households have at least one pet, with dogs being the most common: 52% of households own a dog, and 25% own a cat [68]. According to the Ipsos Observer [69] survey, 73% of respondents (out of a sample of 904) reported owning pets, of which 82% were dogs and 42% were cats. Notably, 74% of respondents considered free-roaming dogs a societal problem, an increase from 55% in 2018. The main concerns included zoonotic disease transmission to humans and animals (46%), risk of bites to people and pets (70%), contamination of public spaces (44%), road accidents (31%), and the hunting of wildlife (13%) [69]. More recently, a 2022 study conducted by the Pontificia Universidad Católica de Chile estimated a national pet population of nearly 12 million animals, including approximately 8.5 million dogs and 3.6 million cats, with 72% of households owning at least one companion animal [70].

## 3. Surgical Sterilization in Dogs: Efficacy, Applications and Limitations

For years, surgical sterilization has been the technique par excellence for the control of unwanted reproduction in pets [71,72,73]. This includes traditional midline-ovariohysterectomy, lateral flank-ovariectomy, castration, early-life gonadectomy, oophorectomy, laparoscopic ovariohysterectomy and ovariectomy, and vasectomy [74]. Gonadectomy is performed primarily on female dogs and is particularly effective in settings where resources are limited or where there is cultural opposition to testicular removal in males [75]. Likewise, sterilization has been used as a therapy for the management of some behavioral problems in dogs, mainly males, notably urine marking, intrasexual aggressiveness and roaming behavior [72,76,77,78,79,80,81,82,83,84] and the prevention of reproductive pathologies such as mammary and testicular tumors [75,81,83,84,85] prevention of pyometra [86], prevention of benign prostatic hypertrophy [85] or prevention of hernias and perineal fistulas [75]. However, some studies show that castration could lead to adverse health and behavioral outcomes, such as the development of orthopedic disorders [75,86,87], and an increased risk of neoplasms, including prostate carcinoma [88], osteosarcoma [89], hemangiosarcoma [75,90], as well as urinary incontinence [90,91]. In humans, surgical sterilization leads to a sustained increase in gonadotropins, particularly follicle-stimulating hormone (FSH), which has been associated with collateral effects such as metabolic alterations, bone loss, and a higher risk of age-related inflammatory diseases [92].

Although gonadectomy can be considered a routine surgical procedure [74], like any other surgery, it requires anesthesia, specialized equipment, appropriate facilities, and sufficient recovery time, and carries the risks associated with surgical interventions [93,94]. Complications may include anesthetic events [95], hemorrhage [96], and issues resulting from inadequate technique, such as ovarian remnant syndrome [74,91,97], infections, dehiscence, urinary incontinence, and ovarian remnant [91].

On the other hand, modelling studies have shown that sustained sterilization over time can reduce dog population density—provided there is no migration of unsterilized animals into the target area. For example, a 20% reduction in population density could be achieved after approximately five years of continuous sterilization campaigns [57]. Dias et al. [44] observed that, even with sterilization of 100% of intact animals annually, it would not be possible to obtain a proportion greater than 86% of dogs after 20 years due to recruitment from neighboring populations.

## 4. Non-Surgical Alternatives for Canine Reproductive Control

The growing need for accessible, reversible, and ethically acceptable complementary alternatives to castration in canine population control has driven the development and implementation of various non-surgical contraceptive strategies. These include chemical castration, vaccination against zona pellucida glycoproteins, the use of synthetic GnRH analogues, and immunocastration through GnRH vaccines. The following sections provide an overview of these methods, discussing the mechanisms of action, advantages, limitations, and current applications.

### 4.1. Chemical Castration

Chemical sterilization involves injecting chemical agents (e.g., calcium chloride or zinc gluconate) into the testicles to induce testicular atrophy, azoospermia, and long-term infertility. Calcium chloride has shown durable sterilizing effects in dogs and rodents [98,99]. Zinc gluconate, although effective, may cause transient local inflammation and testicular tissue necrosis [100,101]. More recent studies support the efficacy of intratesticular zinc gluconate administration in male dogs as a safe, cost-effective, and minimally invasive alternative to surgical castration, although mild post-injection swelling and inflammation have been reported [102,103,104]. Despite being suitable for use in field conditions, chemical sterilization requires specific training and strict handling protocols to ensure both treatment success and animal welfare.

### 4.2. Zona Pellucida Vaccination

Vaccination against zona pellucida (ZP) glycoproteins aims to prevent fertilization by inducing an immune response that produces antibodies capable of blocking sperm-oocyte binding. This method has been widely tested in wildlife species such as white-tailed deer, horses and elephants, with promising and encouraging outcomes [105,106,107]. In companion animals, especially female dogs, the use of porcine ZP vaccines has shown moderate and variable contraceptive efficacy [108,109]. However, the effectiveness is limited by inconsistent antibody production and the need for multiple booster doses to maintain contraceptive effect. Moreover, these vaccines do not suppress estrous behavior and are ineffective in males, reducing the practical utility for population control of free-roaming dogs [110,111].

### 4.3. GnRH Agonists and Antagonists

Another category of non-surgical fertility control involves the use of synthetic GnRH agonists and antagonists. GnRH agonists such as deslorelin initially stimulate, then suppress, the secretion of gonadotropins (LH and FSH). Deslorelin-releasing implants have been successfully used in dogs for temporary and reversible infertility [112,113]. GnRH antagonists like acyline offer immediate suppression of LH and FSH, but require frequent administration and are currently limited in use due to high cost [114]. Both methods offer reversible, hormone-dependent control of reproduction, making them valuable in selected clinical or field contexts.

## 5. Immunocastration as an Alternative to Surgical Castration

Immunocastration involves the use of antigens that induce antibody production against GnRH, disrupting the hypothalamic–pituitary–gonadal axis and leading to suppressed sexual and reproductive function. Compared to surgical sterilization, it offers fewer adverse effects, is cost-effective, and can be applied in large-scale immunization programs [115]. Studies in dogs have demonstrated efficacy in reducing testosterone levels, modifying behaviors such as roaming, aggression, and marking, and achieving reversible fertility suppression [116,117,118].

GnRH is a decapeptide induced at the hypothalamic level and, when transported via the hypothalamic–pituitary portal vein to the adenohypophysis, regulates the release of luteinising hormone (LH) and follicle-stimulating hormone (FSH), both crucial in male and female reproductive physiology [119,120,121]. Immunocastration works by immunoneutralizing GnRH-I, preventing the hormone from binding to receptors on the pituitary gonadotroph cells, thus creating an immunological blockade between the hypothalamus and pituitary gland [118]. This results in decreased secretion of gonadotrophins, inhibition of the hypothalamic–pituitary–gonadal axis, and suppression of gametogenesis and sexual behavior [118,122,123,124,125,126].

GnRH is highly conserved across mammalian species; thus, fertility control technologies targeting the hormone have broad cross-species applicability. Blocking GnRH is used, not only for fertility control, but also to reduce libido, improve meat quality, eliminate carcass odors associated with testosterone, and serve as adjunct treatments for hormone-dependent neoplasms [127]. Immunocastration has been successfully applied in numerous species, including cattle [128,129,130], camelids [131], pigs [119,120,132,133,134,135,136,137,138,139,140] camels [141], equines [126,142,143], rats [144,145,146,147,148], mice [124,149], and sheep [150,151,152,153].

In these studies, a variety of outcomes have been investigated, with particular focus on anti-GnRH antibody (IgG) production, serum testosterone levels, and histopathological changes in gonadal tissue. Table 1 summarizes representative studies on immunocastration conducted in various animal species. Most investigations reported a consistent decrease in testosterone, reduced testicular size and weight, and histological evidence of seminiferous tubule atrophy and impaired spermatogenesis [119,133,134,135]. Kress et al. [139] further confirmed that immunocastration is a reliable and effective method across different production settings.

Research into behavioral changes, particularly in pigs, remains relatively limited and has primarily focused on reductions in sexual (mounting) and aggressive (head-butting, fighting, biting) behaviors, where immunocastration has shown a clear and measurable suppressive effect [119,120,136].

In cattle, immunocastration has demonstrated similar physiological and behavioral effects. Janett et al. [129] reported that immunocastrated young bulls showed elevated antibody titers, decreased testosterone levels, reduced scrotal circumference, and lower physical activity without affecting weight gain. Price et al. [128] observed that head-butting and fighting behavior were significantly reduced in immunocastrated bulls, reaching levels comparable to surgically castrated steers. Siel et al. [154] demonstrated that reduced gonadal function, evidenced by decreased progesterone levels and suppression of estrus, improved productive performance, particularly a better feed conversion efficiency (FCE: 5.58 vs. 5.69 in placebo).

Research in horses has yielded comparable findings. Janett et al. [143] and Turkstra et al. [142] reported reduced circulating testosterone levels, smaller testicular size, suppressed libido, and changes in sperm quality following immunocastration. In donkeys, Rocha et al. [126] found sustained reductions in testosterone concentration, histological degeneration of testicular tissue, and azoospermia in 75% of vaccinated animals.

In sheep, Han et al. [153] found that immunocastration elicited a robust anti-GnRH immune response, decreased testosterone, LH, and FSH levels, and induced testicular atrophy. Gökdal et al. [151] reported that while immunocastration modified reproductive parameters, it did not adversely affect growth performance, carcass quality, or meat quality traits.

Studies in rodents have provided additional insights into the physiological impact of immunocastration. Han et al. [127] and Sáenz et al. [145] both observed significant declines in testosterone and LH levels, testicular atrophy, and impaired spermatogenesis. Furthermore, Han et al. [127] reported enhanced feed conversion and growth rates in immunocastrated rats compared to surgically castrated controls.

Immunocastration is also being explored in wildlife management, with studies demonstrating efficacy in species such as white-tailed deer [106,157], squirrels [158], elk [159], prairie dogs [100], and bison [105]; although these applications are beyond the scope of this review.

Recent studies further strengthen the evidence for immunocastration across species.

In dogs, Siel et al. [117] demonstrated that a new GnRH-based vaccine effectively lowered testosterone levels and reduced undesired behaviors in both experimental and field trials.

In cattle, Huenchullan et al. [160] and Siel et al. [154] confirmed the efficacy of recombinant GnRH vaccines in controlling reproductive functions and improving carcass yields.

In sheep, Zhang et al. [155] found that GnRH immunocastration positively influenced the rumen microbiome profile, improving nutrient utilization and health. Similarly, in pigs, immunocastration protocols were shown to preserve key meat quality traits while effectively reducing boar taint compounds in the Bísaro breed [161].

These findings collectively highlight the versatility and potential of immunocastration as a complementary strategy to surgical castration across a wide range of species, offering advantages not only in reproductive management but also in animal welfare and productivity.

## 6. Contraceptive Strategies and Behavioral Outcomes in Dogs: From Surgical Sterilization to Immunocastration

### 6.1. Sex Steroids, Gonadectomy, and Behavior in Dogs

#### 6.1.1. Hormonal Regulation of Behavior

The hormones produced by the gonads influence a variety of behaviors in vertebrates [162]. During puberty, the central nervous system stimulates the gonads (testes or ovaries) to produce sex hormones, which cause sexual maturation. Neurons in the hypothalamus synthesize and release gonadotropin-releasing hormone (GnRH), which stimulates the production and release of gonadotrophic hormones (FSH and LH) in the anterior pituitary. These, in turn, stimulate male and female gonads to produce sex steroids (testosterone and estradiol, respectively) [163,164].

#### 6.1.2. Surgical Sterilization and Its Physiological Effects

Contraception by surgical sterilization (gonadectomy) is an irreversible procedure that results in the permanent cessation of reproductive function [165]. It consists of the removal of reproductive organs and is used not only to control the population of companion animals but also to confer health benefits, such as reducing the risk of mammary tumors, pyometra, and testicular neoplasms [166]. The most common surgical methods to remove gonads in females are ovariectomy and ovariohysterectomy via the linea alba, flank, or laparoscopic surgery [165], while in males, it is castration [94,167].

#### 6.1.3. Historical Perspective and Sexual Behavior

Since the 1920s, the effect of castration on sexual behavior in various mammalian species has been studied [168]. Hart and Eckstein [77] reported that gonadectomy often results in the complete and immediate elimination of sexual behavior. However, they also noted that non-sexual behaviors may be affected. In a later study, Hart [169] observed that in both dogs and cats, behavioral changes after castration may not appear immediately but rather develop gradually over several weeks. Supporting this, Templin et al. [170] found that approximately 30% of castrated mice exhibited male sexual behavior months after castration, likely due to the persistence of protein effects induced by sex hormones [171] and influenced by previous experience and age at castration [72].

#### 6.1.4. Hormonal Influences on Behavior

Sex hormones such as estrogen in females and testosterone in males regulate key aspects of social and sexual behavior [172]. Castration has been shown to affect mating, erection, and ejaculation [169]. Experimental studies across various species, including rats [173,174], cats [175], dogs [169,176], and rhesus monkeys [177], consistently report reductions in libido and sexual behaviors following castration.

Beyond sexual behavior, sex hormones also influence social interactions and aggressive behaviors within species [178]. Prenatal exposure to androgens plays a crucial role in the development of aggressive tendencies across a wide range of species [179]. Males are generally more aggressive than females, a pattern attributed to the activation effects of androgens [79,180,181,182]. Numerous studies associate castration with a reduction in aggressive behavior, particularly intrasexual aggression among males [72,76,77,78,79,80,81,82,83,84,171,183]. Nevertheless, castration may also increase anxiety and fear responses [77,184,185,186] as sex hormones exert anxiolytic and analgesic effects in various species, including humans [172]. Additionally, Palestrini et al. [187] observed that while castrated male dogs showed reduced mounting behavior, owner-directed aggression showed only a tendency to decrease, and marking behavior did not vary significantly over time. Emotional disturbances such as distress have also been associated with castration [188].

#### 6.1.5. Evidence from Population Studies

Hopkins et al. [76] reported a reduction in aggression toward other males in 60% of castrated dogs, but no change in territorial or fear-induced aggression. McGreevy et al. [189] analyzed behavioral data from 6235 neutered male dogs, finding increased fear and aggression towards strangers and other animals. In females, Podberscek & Serpell [190] noted increased aggression towards children following sterilization. Similarly, Kim et al. [191] observed heightened reactivity in ovariohysterectomised German Shepherd bitches towards unfamiliar people and dogs, while other studies [192,193,194] documented increased aggressiveness towards owners.

Gonadectomy has been shown to significantly reduce mounting and roaming behaviors by between 60% and 90% [76,78,180]. Cannas et al. [195] evaluated owner perceptions in 65 dogs and found no significant changes in behaviors such as play, feeding, or grooming, but observed a significant reduction in mounting and increased reactivity to noise in females.

Territorial marking, particularly using urine, is a sexually dimorphic behavior primarily exhibited by adult males [196,197]. Caffazo et al. [198] described marking as a territorial and competitive behavior among free-roaming dogs. While Beach [196] found that neonatal castration affected marking posture, later studies showed reductions in marking frequency in adult neutered males [76,78,180,183,189,199,200,201] Dogs also use feces and ground scratches for marking, with these signals being more durable and conspicuous than urine [197,202]. However, McGuire [201] did not observe a decrease in marking with feces or scratching after castration.

### 6.2. Sex Steroids, Immunocastration, and Behavior in Dogs

As observed in production and laboratory animals, studies on immunocastration in companion animals (dogs and cats) have mainly focused on immunological and physiological outcomes, while behavioral effects remain less explored [110,203,204,205,206,207,208,209] (see Table 2).

In male cats, Levy et al. [208] reported that three months after vaccination, serum testosterone levels in responsive individuals became undetectable, accompanied by testicular atrophy and reduced spermatogenesis. Later, Levy et al. [110] found that 93% of immunized cats remained infertile during the first year, with effects persisting even for several years.

In Beagle dogs, Basulto et al. [203] demonstrated that high anti-GnRH antibody titers developed six weeks post-vaccination were associated with impaired spermatogenesis and testicular atrophy. Similarly, Jung et al. [116] evaluated various anti-GnRH vaccine formulations, observing that a vaccine comprising canine GnRH conjugated with a T helper cell epitope from canine distemper virus achieved the highest antibody titers and greater suppression of gonadal function.

Walker et al. [204] further confirmed that immunocastration could suppress testosterone and progesterone levels in both male and female dogs. Donovan et al. [206] showed that immunized male dogs developed high anti-GnRH antibody titers, reduced luteinising hormone (LH) and testosterone levels, and significant reductions in testicular volume. Likewise, Liu et al. [207] demonstrated that vaccinated males and females exhibited gonadal atrophy, weight loss, and decreased testosterone and estradiol concentrations.

Behavioral studies following immunocastration have provided additional insights. Bargsted [205] observed that although individual responses varied, male dogs that exhibited aggressive behavior prior to vaccination showed a notable reduction in aggression post-immunization, correlating with decreased testosterone levels. However, no significant differences were found in territorial urine marking before and after treatment. Siel et al. [117] reported that immunized male dogs maintained a high specific immune response up to 150 days, with reductions in gonadal function, sexual behaviors, agonistic interactions, and territorial marking.

Adding to this body of evidence, a recent study by Lin et al. [209] compared the behavioral outcomes of GnRH immunocastration versus surgical castration in rats. Although conducted in a laboratory species, the findings are highly relevant for evaluating immunocastration approaches in companion animals. Both methods effectively suppressed sexual behaviors; however, surgically castrated animals exhibited signs of depression, anxiety, and reduced social interaction, whereas immunocastrated animals maintained emotional stability and normal sociability. No significant differences in learning and memory performance were observed between immunocastrated and intact animals. These results suggest that, beyond reproductive control, immunocastration could offer a welfare-friendly alternative by avoiding the negative emotional side effects associated with surgical sterilization [209].

## 7. Conclusions

Dog overpopulation represents a multifaceted challenge with significant implications for public health, animal welfare, environmental conservation, and biodiversity. Traditional strategies such as mass culling and surgical sterilization, while historically employed, face increasing ethical, social, and logistical limitations. Although surgical gonadectomy remains a highly effective and widely used tool for individual and population-level reproductive control, it has been associated with a range of potential adverse health and behavioral outcomes, including increased risks of orthopedic diseases, urinary incontinence, some cancers, and anxiety-related behaviors.

Immunocastration emerges as a viable and promising alternative to surgical sterilization. By targeting the hypothalamic–pituitary–gonadal axis through GnRH vaccination, immunocastration offers a reversible, minimally invasive, and ethically preferable approach that can be implemented on a large scale. Numerous studies across species, including recent research in dogs, have demonstrated the efficacy of immunocastration in suppressing reproductive function, lowering testosterone levels, and modifying behaviors such as roaming, sexual mounting, and intrasexual aggression, often with fewer emotional side effects compared to surgical castration.

While further long-term studies in companion animals are necessary, the current evidence suggests that immunocastration could become a key element in integrated and sustainable strategies for managing dog populations. It holds potential not only for improving animal welfare but also for addressing broader public health and environmental challenges. Its inclusion in veterinary public health initiatives, alongside responsible ownership programs and educational campaigns, could substantially advance more humane and effective approaches to dog population control.

## Figures and Tables

**Table 1:** Main Outcomes Evaluated in Immunocastration Research by Species.

Author(s) and Publication Year	Species and Sex	Studied Variables
Siel et al. [117]	Dogs (males)	Antibody levels; serum testosterone levels; sperm characteristics; behavioral changes (sexual, agonistic, marking behaviors)
Rydhmer et al. [120]	Pigs (males)	General behavior (walking, sleeping, eating); sexual behavior (mounting); aggressive behaviors (head butts, bites); social behaviors (sniffing, shoving, oral manipulation of another pig’s ears or tail)
Siel et al. [124]	Mice (females and males)	Antibody levels; cytokine expression; histological analysis; serum testosterone levels
Rocha et al. [126]	Equidae (donkeys, males)	Serum testosterone levels; testicular volume; sperm motility; gonadal histological analysis
Price et al. [128]	Cattle (males)	Aggressive behaviors (initiation and participation in fights, head butts)
Janett et al. [129]	Cattle (males)	Antibody levels; serum testosterone levels; body weight; scrotal circumference; physical activity
Noya et al. [130]	Cattle (male)	Growth performance; serum testosterone levels; plasma creatinine levels; plasma urea levels; testicular diameter; subcutaneous fat and skin thickness measurement; aggressive behavior, sexual behavior, physical activity
Einarsson et al. [134]	Pigs (males)	Serum testosterone levels; testicular weight; bulbourethral gland length; sperm morphology; histological study
Brunius et al. [135]	Pigs (males)	Antibody titer; serum testosterone levels; androstenone, skatole and indole levels in adipose tissue; serum oestradiol levels; testicular weight; bulbourethral gland length
Brewster & Nevel [136]	Pigs (males)	Sexual behavior (mounting); aggressive behaviors (threats, fights, head butts)
Pinna et al. [137]	Pigs (males)	Specific characteristics of meat intended for dried ham (sensory characteristics, weight loss due to processing, among others)
Kress et al. [139]	Pigs (males)	Antibody levels; serum testosterone levels; serum cortisol levels; levels of androstenone and skatole in adipose tissue; weight of testes, epididymis, vesicular glands, bulbourethral glands, prostate, and urogenital tract; growth performance
Zoels et al. [140]	Pigs (males)	Antibody levels; serum testosterone levels; testosterone concentration in feces; androstenone and skatole levels in adipose tissue; growth performance; penile injuries
Ghoneim et al. [141]	Dromedary camels (males)	Serum testosterone levels; testicular volume; semen characteristics; sexual behavior (libido)
Turkstra et al. [142]	Equidae (ponies, males)	Antibody levels; serum testosterone levels; sperm motility; gonadal histological analysis; testicle size
Janett et al. [143]	Equidae (foals, males)	Antibody levels; serum testosterone levels; sperm quality; scrotal diameter; sexual behavior (libido)
Fang et al. [149]	Mice (males)	Antibody levels; gonadal histological analysis; testicular weight
Siel et al. [154]	Cattle (females)	Antibody levels; serum progesterone levels; estrus presentation; reproductive behavior; productive parameters (weight gain)
Zhang et al. [155]	Sheep (males)	Antibody levels; serum testosterone levels; rumen microbiome profile; rumen metabolite profile; body weight gain
Werner et al. [156]	Pigs (males)	Gonadal histological analysis; fattening performance; agonistic behavior (pushing, biting, and mounting); animal welfare (penis scratches and injuries); serum testosterone levels; androstenone and skatole levels in adipose tissue

**Table 2:** Summary of the main variables studied in immunocastration studies on companion animals, in alphabetical order by species and year of publication.

Author(s) and Year of Publication	Species and Sex	Studied Variables
Levy et al. [110]	Cats (females)	Antibody levels; indicators of reproductive success; serum estradiol and progesterone levels
Jung et al. [116]	Dogs (males)	Antibody levels; gonadal histological analyses
Siel et al. [117]	Dogs (males)	Antibody levels; testosterone serum tests; spermiogram; agonistic, sexual, territorial marking and affiliative behavior
Basulto et al. [203]	Dogs (males)	Antibody levels; spermiogram; gonadal histological analyses
Walker et al. [204]	Dogs (males and females)	Antibody levels; serum levels of testosterone and progesterone
Bargsted [205]	Dogs (males)	Endocrine profile; antibody levels; gonadal histological analysis; aggressive behavior, sexual and territorial marking.
Donovan et al. [206]	Dogs (males)	Endocrine profile; antibody levels; testicular volume
Liu et al. [207]	Dogs (males and females)	Antibody levels; serum testosterone and estradiol levels; gonadal histological analysis; weight and gonadal size
Levy et al. [208]	Cats (males)	Antibody levels; serum testosterone levels; scrotal size; gonadal histological analyses

## Data Availability

No new data were created or analyzed in this study. Data sharing is not applicable to this article.
